# Adenovirus infection associated with focal segmental glomerulosclerosis following autologous hematopoietic stem cell transplantation

**DOI:** 10.1007/s13730-026-01110-9

**Published:** 2026-05-20

**Authors:** Yasser Isaac Arana-Escandón, Paula Eliana Ramírez-Arboleda, Lina María Gaviria-Jaramillo, Mauricio Andrés Álzate-Arias, Oliver Gerardo Perilla-Suarez, María Ximena Lagos-Buitrago, Joaquín Roberto Rodelo-Ceballos, Mayra Alejandra Estacio, Alejandra Taborda-Murillo

**Affiliations:** 1 Present Address: Departamento de Medicina Interna, Facultad de Medicina, Universidad de Antioquia UdeA, Calle 70 No. 52-21, Antioquia Medellín, Colombia; 2 Hematology Service, Hospital Universitario San Vicente Fundación, Medellín, Colombia; 3 Nephrology Service, Hospital Universitario San Vicente Fundación, Medellín, Colombia; 4 Present Address: Nefrología, Departamento de Medicina Interna, Facultad de Medicina, Universidad de Antioquia UdeA, Calle 70 No. 52-21, Antioquia Medellín, Colombia; 5 Present Address: Departamento de Patología, Facultad de Medicina, Universidad de Antioquia UdeA, Calle 70 No. 52-21, Medellín, Colombia

## Abstract

Disseminated adenovirus infection is a rare but often fatal complication of hematopoietic stem cell transplantation (HSCT), particularly uncommon in the autologous setting. We report the case of a 59-year-old man with stage IVB diffuse large B-cell lymphoma who underwent autologous HSCT after multiple lines of therapy. Following conditioning regimen, he developed persistent fever, mucositis, dysuria, and gross hematuria, with imaging showing bilateral pyelonephritis, ureteritis, and cystitis. Despite broad-spectrum antibiotics, symptoms persisted. Quantitative PCR revealed rapidly rising adenovirus viremia (> 1,000,000 copies/mL), and renal biopsy demonstrated viral cytopathic changes on light microscopy together with focal segmental glomerulosclerosis (FSGS). The patient progressed to fulminant oliguric renal failure and died before dialysis could be initiated. This case highlights a rare presentation of adenovirus infection with renal involvement consistent with adenovirus-associated nephropathy and concurrent FSGS after autologous HSCT, highlighting the potential convergence of viral, neoplastic, and transplant-related mechanisms in catastrophic renal injury.

## Introduction

Disseminated adenovirus infection is an uncommon but severe complication of hematopoietic stem cell transplantation (HSCT). It is most frequently described in the setting of allogeneic HSCT, particularly in pediatric patients or in those with profound immunosuppression, and is associated with high morbidity and mortality [1]. In autologous HSCT, by contrast, disseminated adenovirus disease is less frequent [2]. Renal involvement typically manifests as hemorrhagic cystitis or, less frequently, as tubulointerstitial nephritis, whereas glomerular injury due to adenovirus is scarcely documented [3].

Focal segmental glomerulosclerosis (FSGS) represents another unusual renal lesion in the post-transplant setting. While minimal-change disease and membranous nephropathy are the predominant glomerulopathies associated with hematological malignancies and transplantation, FSGS has been described only rarely [4]. Potential mechanisms include paraneoplastic phenomena, immune dysregulation after transplantation, exposure to nephrotoxic agents, and, more recently, viral infections such as HIV, HBV, parvovirus B19, and CMV [5].

Here we report a case of fulminant renal failure in a patient with diffuse large B-cell lymphoma who underwent autologous HSCT and subsequently developed adenovirus infection with severe genitourinary involvement. Renal biopsy revealed focal segmental glomerulosclerosis together with tubular viral cytopathic changes on light microscopy. This case underscores the rarity of this association and highlights the potential convergence of viral infection, underlying malignancy, and transplant-related immune dysregulation leading to catastrophic renal injury.

## Case report

We present the case of a 59 years old male patient with a medical history of a mass in the hard palate. Biopsy revealed an aggressive large B-cell non-Hodgkin lymphoma (NHL), positive for CD20 and CD7, strongly positive for PAX-5, with a Ki-67 index of 70%, and a confirmed C-MYC rearrangement by fluorescence in situ hybridization (FISH); negative for BCL-2 and BCL-6. The disease was classified as stage IVB due to additional involvement of lymph nodes at the base of the neck, mediastinum, bilateral pulmonary hila, and infradiaphragmatic areas, including the gastrohepatic space.

The patient had no history of hypertension, diabetes mellitus, obesity, or prior kidney disease. Baseline renal function before transplantation was normal (serum creatinine 0.86 mg/dL), and there was no documented proteinuria prior to HSCT.

Initial treatment with six cycles of R-CHOP failed due to new mediastinal involvement. The patient then received two cycles of salvage therapy with R-ICE, during which disease progression was observed. Subsequently, treatment was switched to the Pola-BR protocol and 20 sessions of VMAT radiotherapy to the nasopharynx (total dose: 40 Gy). PET-CT evaluation revealed Deauville X due to new lesions in both pulmonary hilum, prompting consideration for autologous hematopoietic stem cell transplantation.

The patient was admitted for autologous transplant protocol initiation. The transplant comorbidity index (HCT-CI) was 1, and following the hematology team’s assessment, mobilization with filgrastim was initiated. The first apheresis yielded 2,581,189 CD34 + cells/kg. Conditioning was performed with Cyclophosphamide, Carmustine, and Etoposide (CBV regimen).

Two days post-infusion, the patient developed febrile neutropenia with multiple episodes of diarrhea. A multiplex gastrointestinal PCR panel detected Escherichia coli and ruled out Clostridioides difficile. Blood cultures were positive for Escherichia coli and Klebsiella spp. with CTX-M gene detection. The patient was treated with meropenem, resulting in clinical improvement after seven days and negative follow-up blood cultures. Engraftment occurred on day + 16 and + 17 for platelet and granulocytic grafts, respectively.

**Fig. 1:**
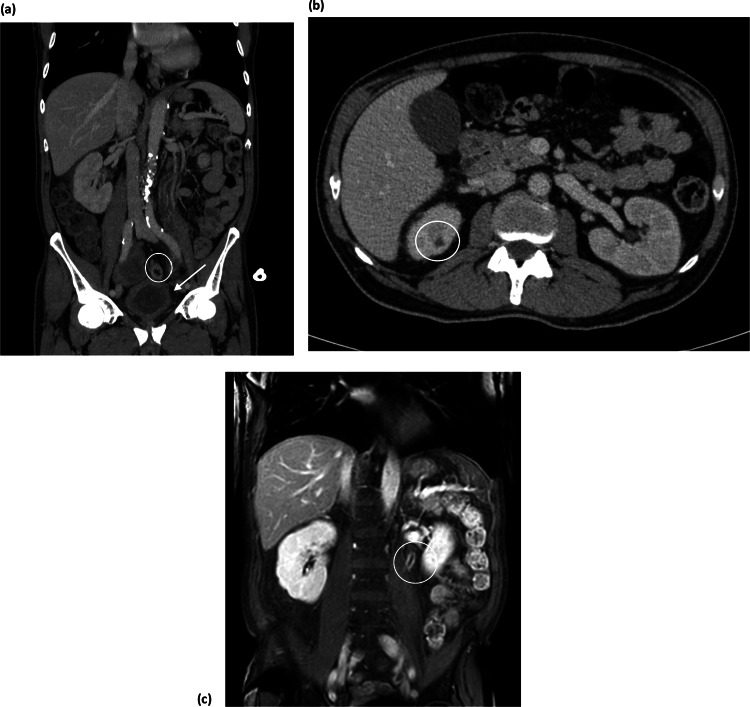
Abdominal imaging demonstrating extensive involvement of the urinary tract. **a **Coronal view of contrast-enhanced abdominal computed tomography: The circle indicates thickening of the left ureter. The arrow indicates diffuse bladder wall thickening. **b **Axial view of contrast-enhanced abdominal computed tomography: The circle shows a developing perirenal abscess. **c **Coronal view of contrast-enhanced T1-weighted sequence with subtraction in the venous phase on urinary tract MRI: The circle indicates diffuse ureteral thickening

On day + 13 post-infusion, the patient experienced a recurrence of fever, with severe mucositis and new-onset dysuria as the only localizing symptoms. Meropenem was reinitiated, and amikacin plus vancomycin were added for broad-spectrum coverage, including gram-positive cocci. Urinalysis showed sterile pyuria and a negative urine culture. Despite neutrophil recovery, fever persisted, and the patient developed gross hematuria. A contrast-enhanced abdominal CT showed bilateral pyelonephritis, a possible abscess in the upper pole of the left kidney, bilateral ureteritis, and cystitis—findings later confirmed by MRI (Fig. 1).

Given the persistence of fever despite broad-spectrum antibiotics and the radiologic evidence of extensive urinary tract involvement, a viral etiology was suspected. Serum viral load testing for BK virus (BKV), adenovirus, and cytomegalovirus (CMV) was requested, along with urinary cytology for BKV.

**Fig. 2:**
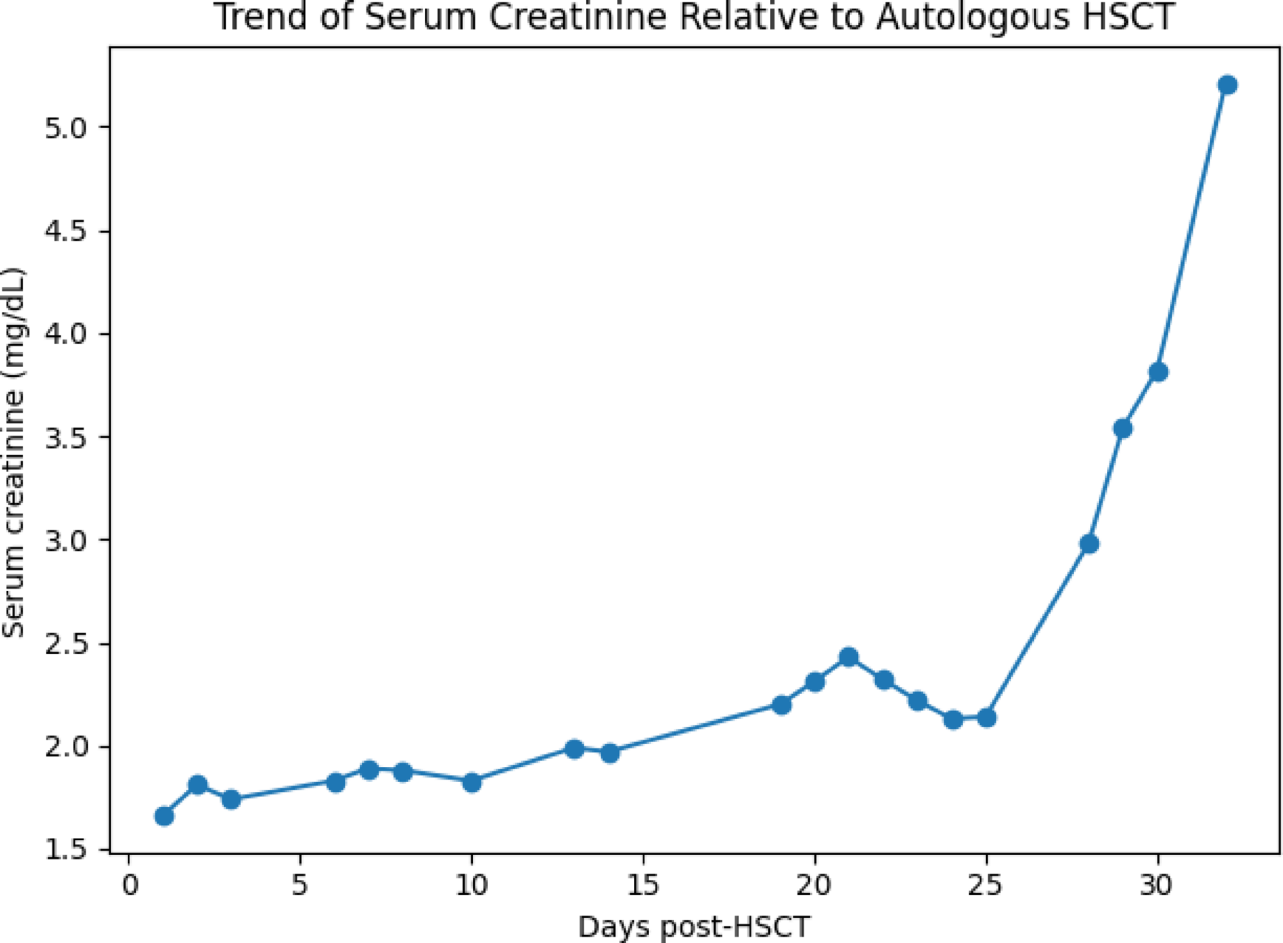
Trend of serum creatinine levels (mg/dL) over time relative to autologous hematopoietic stem cell transplantation

Renal function progressively deteriorated after transplantation, with serum creatinine rising from a baseline of 0.86 mg/dL prior to HSCT to 1.99 mg/dL at the onset of gross hematuria, and further increasing to 5.21 mg/dL despite supportive care. The temporal evolution of serum creatinine in relation to key clinical events is shown in Fig. 2. Urinalysis and quantification at the time of deterioration revealed proteinuria and hematuria, but no casts were observed. Quantification confirmed nephrotic-range proteinuria of 6,396 mg/24 h. A renal biopsy was therefore performed.

**Fig. 3:**
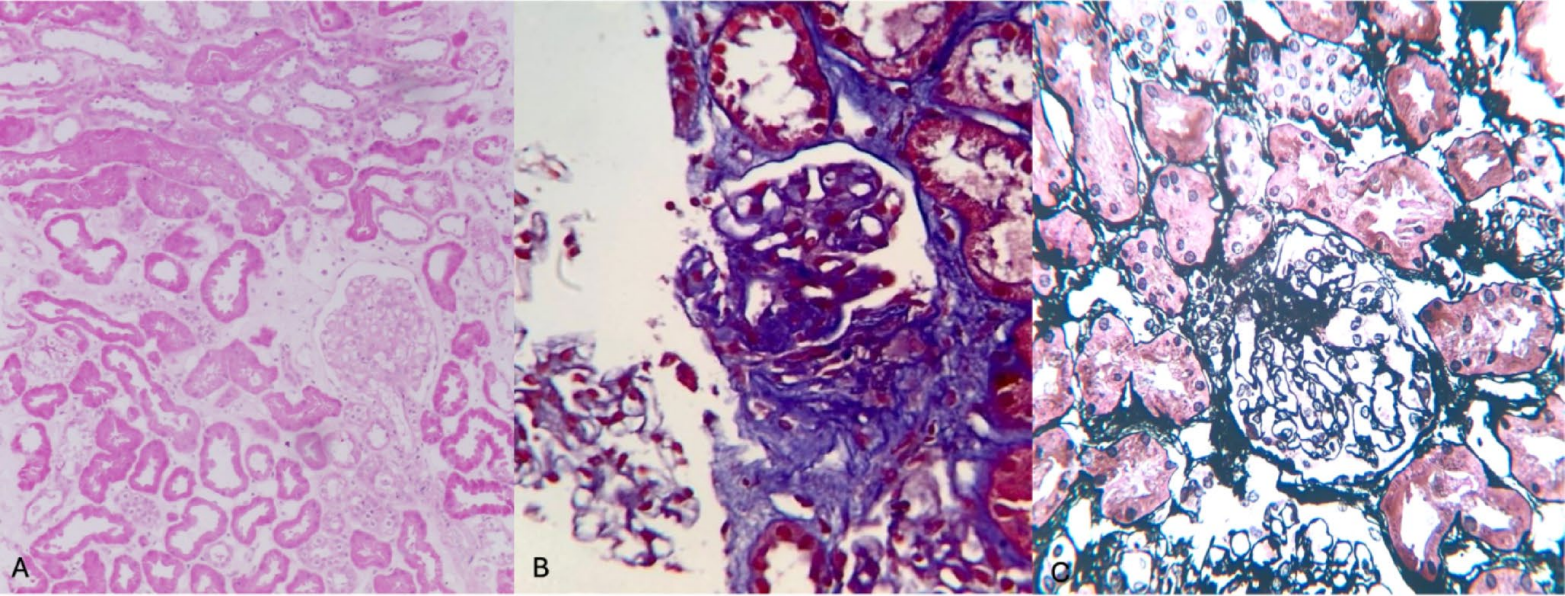
Renal biopsy demonstrating focal segmental glomerulosclerosis. **A** Low-magnification view showing preserved overall renal architecture with tubular and interstitial compartments (H&E, low power). **B** Glomerulus with segmental sclerosis (Masson’s trichrome stain, high power). **C** Glomerulus with perihilar sclerosis highlighted by silver methenamine stain (high power).

Histopathological examination revealed 26 glomeruli, with global sclerosis in 4% and segmental sclerosis in 4% (Fig. 3). The remaining glomeruli showed podocyte hypertrophy without mesangial or endocapillary hypercellularity. Tubules displayed epithelial necrosis and viral cytopathic nuclear changes.

**Fig. 4:**
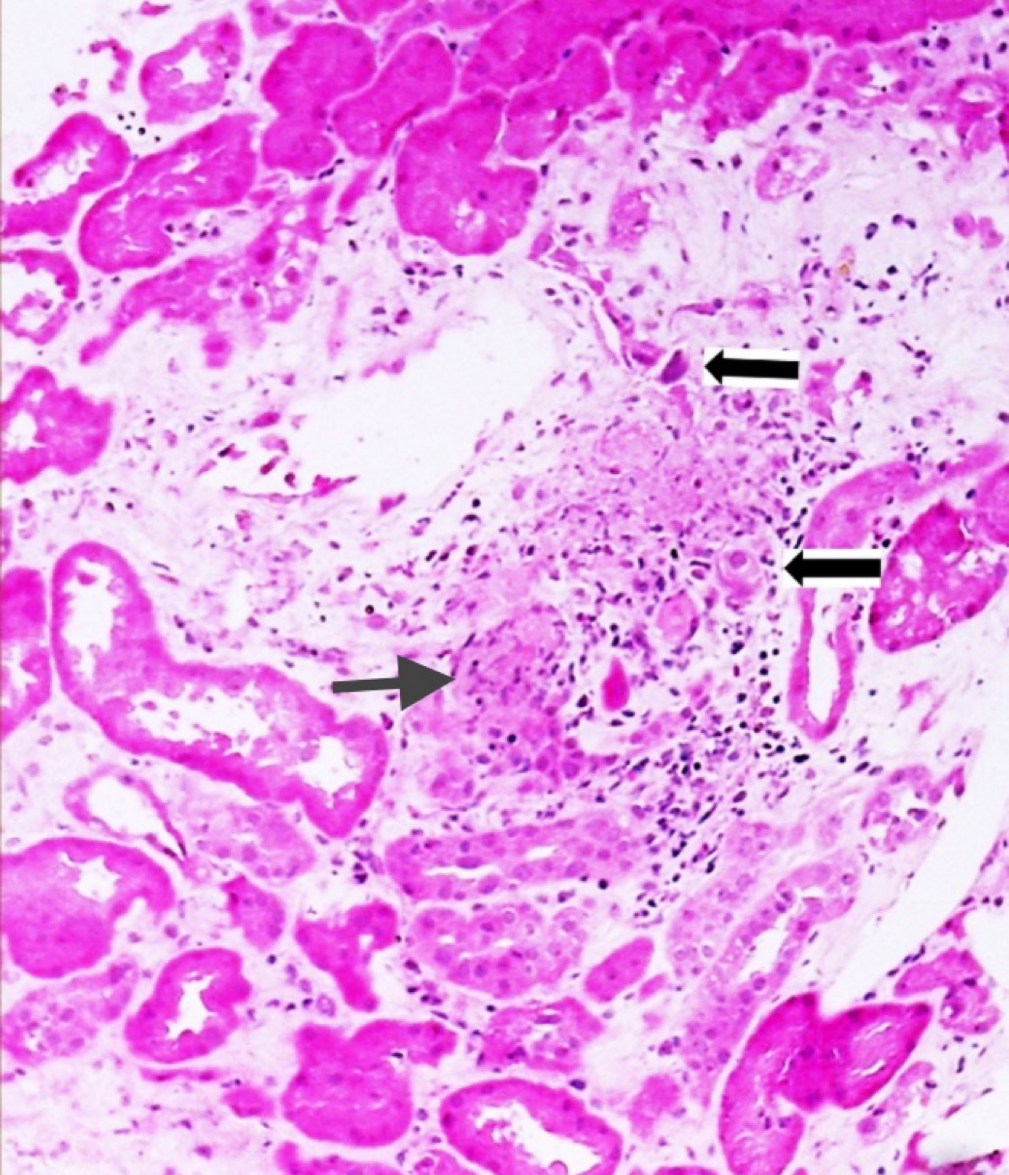
Renal biopsy: cytopathic changes due to adenovirus. Tubular epithelial cells with nuclear enlargement and viral cytopathic inclusions with a smudged basophilic appearance, associated with focal epithelial necrosis and interstitial inflammation *(H&E, X400)*

Cytopathic changes included basophilic intranuclear inclusions with a smudged appearance, more prominent in distal tubules, and detached infected cells within the tubular lumina (Fig. 4). Minimal interstitial fibrosis and tubular atrophy (< 5%) and mild arterial changes were noted, without evidence of thrombotic microangiopathy. Immunofluorescence was negative for immune deposits, and SV40 immunohistochemistry ruled out polyomavirus. Overall, the findings were consistent with focal segmental glomerulosclerosis together with renal involvement associated with adenovirus infection, in the appropriate clinical and virological context.

Adenovirus viremia was initially detected at 159,547 copies/mL. Over the following three weeks, viral loads rose rapidly, exceeding 1,000,000 copies/mL. Treatment with cidofovir plus probenecid was considered; however, cidofovir was not administered due to rapid clinical and renal deterioration, which precluded its initiation. The patient developed progressive renal failure with creatinine rising to 5.21 mg/dL, accompanied by oliguria, hyperkalemia, and uremic encephalopathy. He required urgent initiation of dialysis; however, he died before renal replacement therapy could be started.

## Discussion

Adenovirus infection is an uncommon but severe complication in hematopoietic stem cell transplantation (HSCT) [6]. Its incidence is highest in pediatric and allogeneic recipients, particularly with profound T-cell suppression, while autologous HSCT cases are rarely described. In autologous recipients, disease is usually limited to hemorrhagic cystitis, and disseminated infection with renal involvement is exceptional [2]. In our patient, renal involvement consistent with adenovirus-associated nephropathy in the setting of high-level viremia and catastrophic renal failure represents an unusual and severe manifestation.

Adenovirus infection after HSCT has been characterized in large cohorts. Inamoto et al. reported a cumulative incidence of adenovirus disease of 0.49% in adults compared to 2.99% in children, and of 0.18% in autologous versus 1.52% in allogeneic recipients [2]. Among 22,503 adult autologous HSCT recipients, only 115 developed adenovirus disease, with genitourinary involvement being the most frequent manifestation—consistent with our case. Similarly, Moret et al. observed adenovirus as the cause of acute respiratory infection in only 0.6% of autologous HSCT patients, highlighting its rarity in this setting [7]. In contrast, Styczynski et al. described that in allogeneic HSCT recipients, adenovirus most commonly presented as viremia (62.6%), followed by gastrointestinal involvement (17.9%), cystitis (3.7%), and pneumonia (4.9%) [8]. In our patient, disseminated infection was confirmed by persistent high-level viremia together with renal disease.

A major limitation of this case is the absence of adenovirus-specific immunohistochemistry, in situ hybridization, or electron microscopy on renal tissue. Therefore, definitive histopathologic confirmation of adenovirus infection within the kidney cannot be established. Nevertheless, the combination of characteristic viral cytopathic changes on light microscopy, markedly elevated adenovirus viremia, compatible clinical presentation, and exclusion of alternative viral etiologies supports a clinicopathologic diagnosis of adenovirus-associated renal involvement.

In our patient, the presence of focal segmental glomerulosclerosis is particularly remarkable given its rarity in this setting. Most cases have been described in allogeneic settings, often related to graft-versus-host disease or calcineurin inhibitor exposure. Reports following autologous HSCT are extremely limited, and the underlying mechanisms remain incompletely understood. Hypotheses include immune reconstitution abnormalities, circulating permeability factors, or podocyte injury triggered by drugs or infections [5]. In this patient, the presence of focal segmental glomerulosclerosis should be interpreted with caution. Given the absence of traditional risk factors for secondary FSGS and the lack of prior proteinuria, the lesion most likely reflects a secondary or adaptive process in a complex clinical setting. Potential contributing mechanisms include immune dysregulation following autologous transplantation, paraneoplastic effects related to lymphoma, and a possible viral trigger in the context of disseminated adenovirus infection.

Viral infections have been increasingly recognized as potential triggers of FSGS, most notably HIV but also hepatitis B virus, parvovirus B19, cytomegalovirus, and Epstein–Barr virus [9]. Mechanisms include direct podocyte infection, viral protein toxicity, and immune-mediated injury [10]. Although adenovirus has not been firmly established as a cause of FSGS, the temporal association in this case raises the possibility that disseminated adenovirus infection contributed to podocyte damage and the emergence of FSGS.

Another potential contributor is the underlying lymphoma. Glomerular disease is a recognized paraneoplastic phenomenon in hematologic malignancies, particularly Hodgkin lymphoma, where minimal-change disease predominates [11]. In non-Hodgkin lymphoma, more diverse lesions, including membranous nephropathy and, rarely, FSGS, have been reported [12, 13]. In our patient, diffuse large B-cell lymphoma represents a possible predisposing factor; however, the temporal association of renal deterioration with disseminated adenovirus infection suggests that the viral process played a central role, with the lymphoma acting as a background risk factor rather than the immediate trigger.

Therapeutic options for adenovirus infection in adults remain limited. Cidofovir, usually administered with probenecid to reduce nephrotoxicity, is the most widely recommended antiviral; however, its use is frequently restricted by the very renal dysfunction that adenovirus infection precipitates [14]. Alternative approaches, such as brincidofovir, adoptive T-cell therapy, and intravenous immunoglobulin, have been explored, but data in adult HSCT recipients are scarce and largely confined to small series or case reports [15]. Consequently, despite early recognition and initiation of available therapy, reported mortality rates for disseminated adenovirus infection remain close to 50%.

Disseminated adenovirus infection after autologous HSCT is uncommon but should remain in the differential diagnosis of persistent fever with genitourinary manifestations. This case demonstrates an exceptional association of adenovirus nephropathy with focal segmental glomerulosclerosis, likely reflecting a multifactorial pathogenesis involving viral injury, paraneoplastic processes, and transplant-related immune dysregulation. The fulminant renal deterioration observed emphasizes the critical role of renal biopsy in guiding diagnosis, and the limitations of current antiviral options in the setting of advanced kidney injury. Greater awareness of this rare complication may facilitate earlier recognition, closer monitoring, and future strategies aimed at improving outcomes in this high-risk population.

## Declarations

### Human and/or animals participants

All procedures performed in studies involving human participants were in accordance with the ethical standards of the institutional and/or national research committee and with the 1964 Helsinki declaration and its later amendments or comparable ethical standards. This article does not contain any studies with animals performed by any of the authors.
